# Anti-survivin effect of the small molecule inhibitor YM155 in RCC cells is mediated by time-dependent inhibition of the NF-κB pathway

**DOI:** 10.1038/s41598-018-28213-3

**Published:** 2018-07-06

**Authors:** Mei Yi Sim, John Shyi Peng Yuen, Mei Lin Go

**Affiliations:** 1 Department of Pharmacy, National University of Singapore, 18 Science Drive 4, Singapore, 117543 Republic of Singapore; 2 Department of Urology, Singapore General Hospital, 20 College Road, Singapore, 169856 Republic of Singapore

## Abstract

Constitutive activation of the NF-κB signaling cascade is associated with tumourigenesis and poor prognosis in many human cancers including RCC. YM155, a small molecule inhibitor of survivin, was previously shown to potently inhibit the viability of immortalized and patient derived renal cell carcinoma (RCC) cell lines. Here we investigated the role of NF-κB signaling to the anti-cancer properties of YM155 in RCC786.0 cells. YM155 diminished nuclear levels of p65 and phosphorylated p65 and attenuated the transcriptional competencies of the p65/p50 heterodimers. Accordingly, we found that YM155 diminished the transcription of NF-κB target gene survivin. Events that led to the interception of the nuclear translocation of p65/p50 were the activation of the deubiquinating enzyme CYLD by YM155, which led to the inhibition of IKKβ, stabilization of IκBα and retention of NF-κB heterodimers in the cytosol. Importantly, the suppressive effects of YM155 were time-dependent and observed at the 24 h time point, and not earlier. TNF-α, a stimulator of NF-κB signaling did not affect its inhibitory properties. The ability of YM155 to intercept a major transcriptional pathway like NF-κB, would have important ramifications on the pharmacodynamics effects elicited by this unusual molecule.

## Introduction

Renal cell carcinoma (RCC) is the most lethal urologic malignancy. Approximately one in three patients with RCC exhibit visceral metastasis^1^ at the time of diagnosis while half of the remaining patients would eventually develop distant metastases after surgery^2^. The clear cell histological variant is the most common manifestation of renal cell carcinoma and has a pathology that is unique among other RCC subtypes in its association with the mutated von Hippel Lindau (*VHL*) tumour suppressor gene.

**Figure 1:**
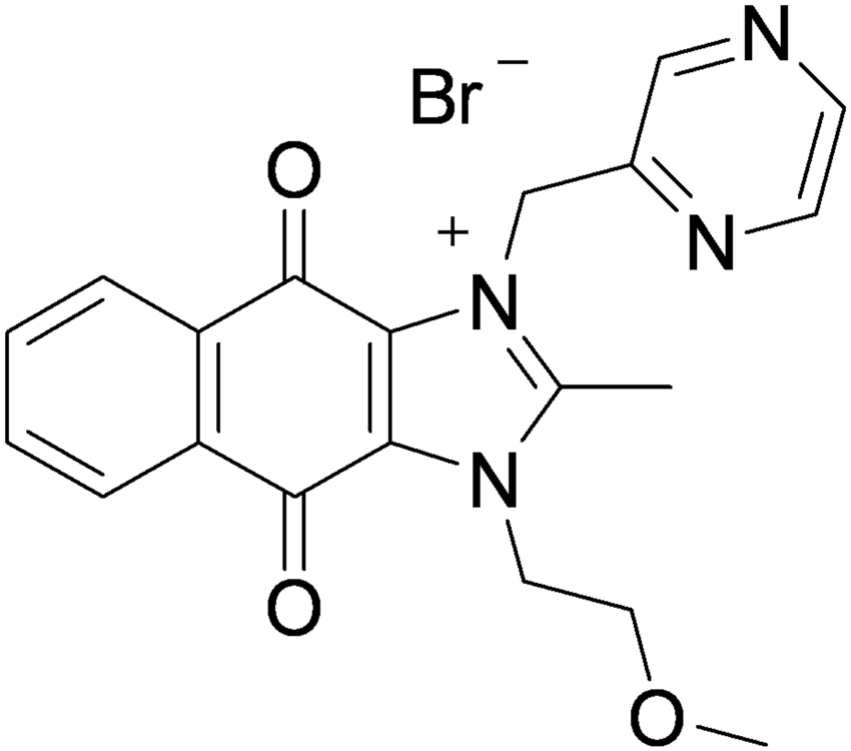
Structure of YM155 [1-(2-methoxyethyl)−2-methyl-4,9-dioxo-3-(pyrazin-2-ylmethyl)−4,9-dihydro-1H-naphtho[2,3-d]imidazol-3-ium bromide].

The dioxonaphthoimidazolium analog and survivin suppressant YM155 potently inhibits cell growth and induces apoptosis in several human cancer cell lines and xenograft models^3–6^ (Fig. 1).

While YM155 suppresses survivin levels in cancer cells, as confirmed in several investigations^7–10^, there is growing consensus that other oncogenic proteins or pathways are involved in its potent apoptogenic effects^11–13^. Earlier, we have shown that YM155 exhibited consistent nanomolar potencies across a panel of immortalized and patient derived RCC cell lines, independently of their VHL status and their survivin expression levels^13^. Analysis of the genes that were differentially expressed in YM155-treated RCC786.0 (a VHL negative cell line derived from a human primary clear cell adenocarcinoma) revealed prominent clustering within the p53 connection pathway. We followed up on three genes that were relevant to RCC pathogenesis and have not been implicated as targets of YM155, namely CYLD (cylindromatosis, a deubiquitinating enzyme that negatively regulates NF-κB transcription activity), FOXO1 (a tumour suppressor that mediates cell cycle arrest and promotes apoptosis) and ID1 (an inhibitor of DNA binding that suppresses transcriptional activation of HLH proteins which leads to tumour growth and angiogenesis)^13^. Collateral evidence from both mRNA and protein analyses revealed that YM155 downregulated ID1 expression but stimulated CYLD and FOXO1. The activation of CLYD by YM155 was of particular interest because YM155 has been reported to intercept the NF-κB pathway in a non-small cell lung cancer cell line^14^. The NF-κB transcriptional network plays a pivotal role in regulating cell proliferation, invasion and apoptosis. Briefly, NF-κB is sequestered in an inactive state within the cytosol by inhibitory IκBs which mask the DNA binding ability and hence transcriptional competency of NF-κB. In the presence of activating stimuli, the IκBs are phosphorylated by IκB kinases (IKKs) and directed to the proteasome for degradation. This allows the liberated NF-κB (mainly p65/p50 heterodimers) to be phosphorylated and transported to the nuclei where they recruit chromatin remodelling factors on the NF-κB promoters for activation of target genes.

The reciprocity between the p53 and NF-κB signaling pathways which are known to modulate each other’s activities, is widely recognized^15–17^. While the effects of YM155 on cell cycle progression and cell death, which are outcomes of p53 regulation, are well established, less is known of its interactions with the NF-κB signaling pathway. These knowledge gaps restrict the rational deployment of YM155 singly or in combination with other agents for cancer chemotherapy. Here we demonstrate that YM155 caused a time dependent attenuation of NF-κB signaling in RCC786.0 cells, primarily by intercepting the nuclear translocation of the p65 subunit and hence curtailing its transcriptional activity.

## Results

### YM155 has a time-dependent biphasic effect on the binding of p65/p50 heterodimers to its DNA consensus sequences

RCC786.0 cells lack a functional pVHL due to mutation of the *VHL* gene but they possess wild type p53. We reconfirmed our earlier findings that YM155 potently inhibited the proliferation of RCC7860.0 cells at nanomolar concentrations (growth inhibitory IC_50_ 40 nM) (Supplementary Figure 1: DRC). To determine if YM155 intercepted the binding of NF-κB dimers (of which p65/p50 is the most prevalent) to their consensus sequences, we treated RCC786.0 cells with varying concentrations of YM155 (20 nM to 160 nM) at two time points (6 h and 24 h). The experiments were carried with or without the presence of TNF-α, a stimulator of the NF-κB pathway.

**Figure 2:**
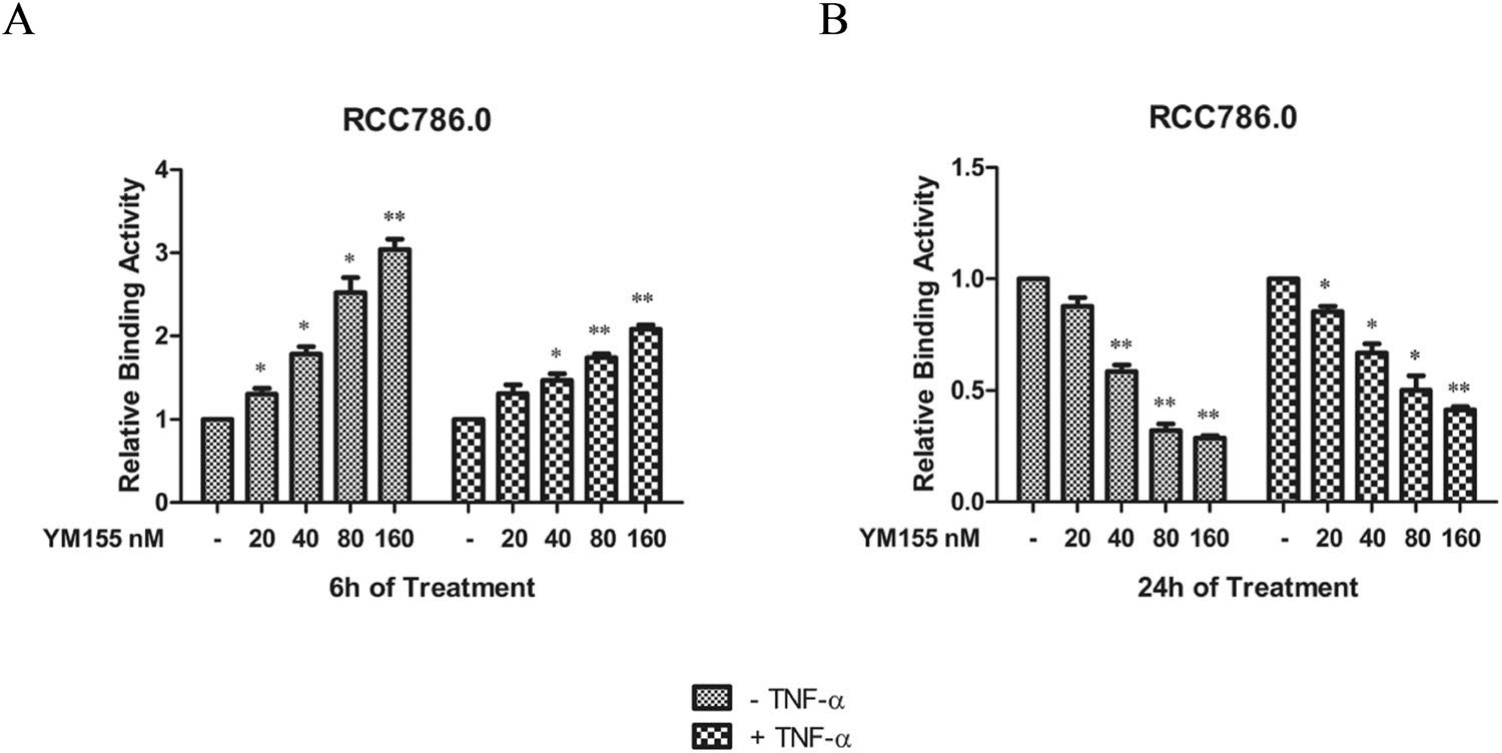
Effects of YM155 on the DNA binding activity of p65 NF-κB. RCC786.0 was treated with 0 nM to 160 nM of YM155 for 6 h and 24 h in the absence or presence of TNF-α stimulation. The nuclear fraction was extracted and used in a p65 NF-κB transcription factor binding assay. (**A**) YM155 increased binding affinity of p65 NF-κB to its consensus sequence after 6 hours. (**B**) YM155 decreased DNA binding affinity of p65 NF-κB to its consensus sequence after 24 hours. Cells that were not subjected to TNF-α stimulation showed a greater change in DNA binding activity. *p < 0.05 and **p < 0.01 from its control.

We found that YM155 promoted the binding of p65 NF-κB subunit to its DNA consensus sequences at the 6 h time point. Its effects were concentration dependent and more pronounced (by approximately 3 fold) in the absence of TNF-α (Fig. 2A). Intriguingly, YM155 induced an opposite effect at the longer 24 h time point where it attenuated the binding of p65 NF-κB subunit to the DNA consensus sequences (Fig. 2B). The losses in binding affinity were concentration dependent and as before, more pronounced in the absence of TNF-α.

We see here that YM155 exerts a biphasic, time dependent effect on the binding of p65 to its consensus sequence. An early increase in binding affinity (6 h) was followed by a decline at the longer 24 h time point. Both effects were concentration dependent and more pronounced in cells that were not stimulated by TNF-α.

### Transcription activity of NF-κB was disrupted by YM155

**Figure 3:**
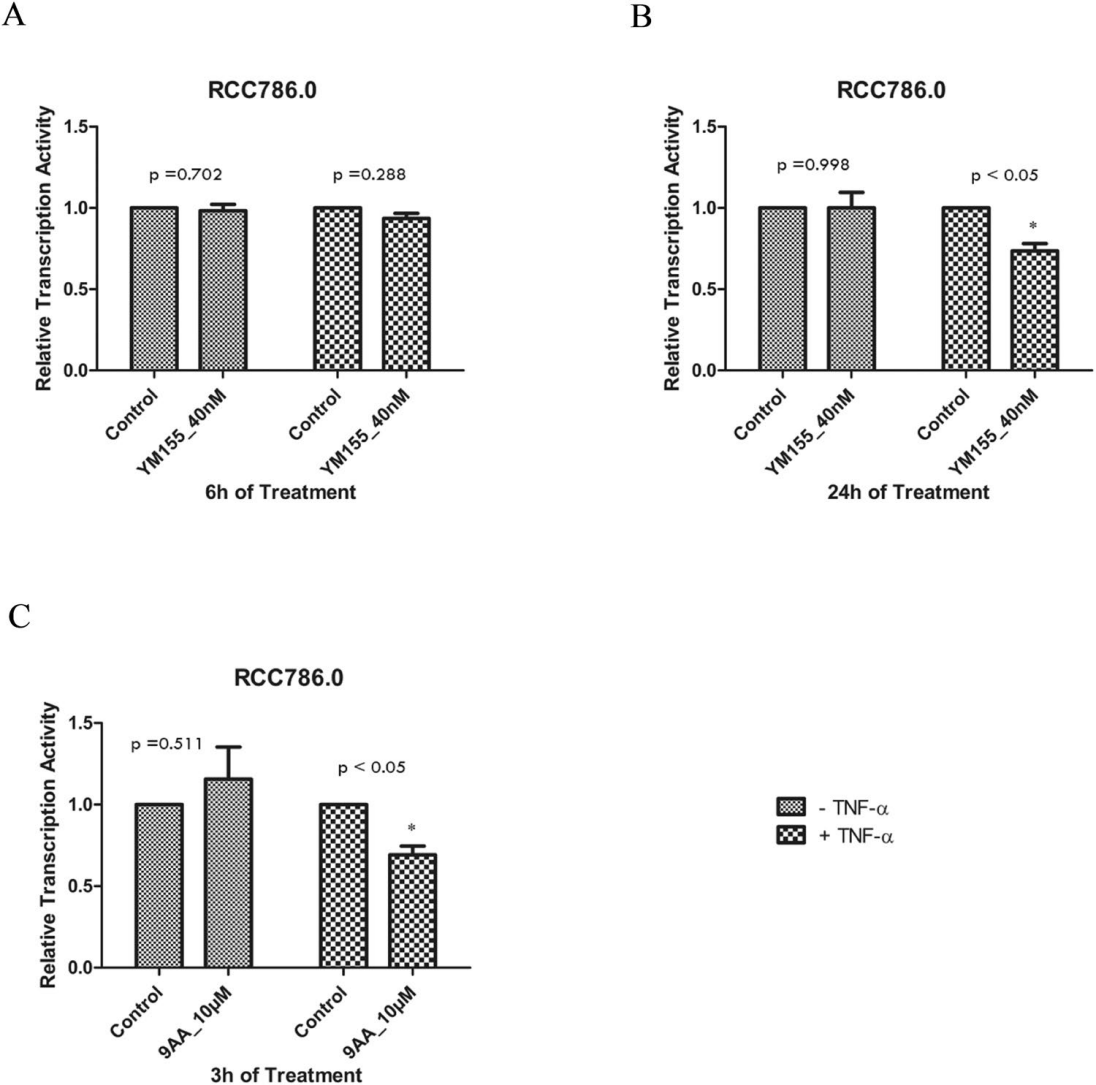
Effects of YM155 and 9-aminoacridine (9AA) on the transcription activity of NF-κB. RCC786.0 was treated with 40 nM of YM155 for 6 h and 24 h in the absence or presence of TNF-α stimulation. In a luciferase reporter assay, (**A**) YM155 did not affect the transcription activity of NF-κB at 6 h. (**B**) YM155 reduced the transcription activity of NF-κB to 73% of the control in the presence of TNF-α at 24 h. A known inhibitor of NF-κB dependent transcription, 9AA was used as positive control. (**C**) 9AA reduced the transcription activity of NF-κB to 69% of the control when subjected to a treatment concentration of 10 µM for 3 h in the presence of TNF-α. *p < 0.05 from its control.

As the binding of NF-κB dimers to their consensus sequences would activate transcription of NF-κB controlled genes, we anticipated YM155 to effect an initial increase in transcriptional activity followed by a subsequent decrease, in line with its biphasic effects on DNA binding. To investigate, RCC786.0 cells were transiently transfected with a luciferase reporter gene whose expression was regulated by NF-κB. These cells were then exposed to YM155 (40 nM) for 6 h and 24 h, in the absence or presence of TNF-α. Similar experiments were carried out with 9-aminoacridine (9AA, 10 uM), a known inhibitor of NF-κB dependent transcription^18,19^. As shown in Fig. 3C, 9AA suppressed transcription (by approximately 30%) within 3 h but only in the presence of TNF-α. In the case of YM155, we did not detect any change in transcriptional activity after 6 h, with or without TNF-α (Fig. 3A). There was however a decrease in transcriptional activity (by ~27%) at the 24 h time point, but only in the presence of TNF-α (Fig. 3B), similar to the positive control 9AA. We posit that the basal transcription activity in unstimulated cells may have been too low at these time points for inhibition of YM155 to be detected by the luciferase reporter assay. Taken together, we did not find any concordance between YM155-induced DNA binding and transcriptional activity at the early time point, but the diminished binding of p65 to its consensus sequences did translate to reduced transcription in YM155 treated cells at the late 24 h time point and in the presence of TNF-α.

### YM155 has a time dependent effect on the cytosol to nuclear translocation of p65 but did not increase phosphorylated p65 levels in nucleus at either time point

**Figure 4:**
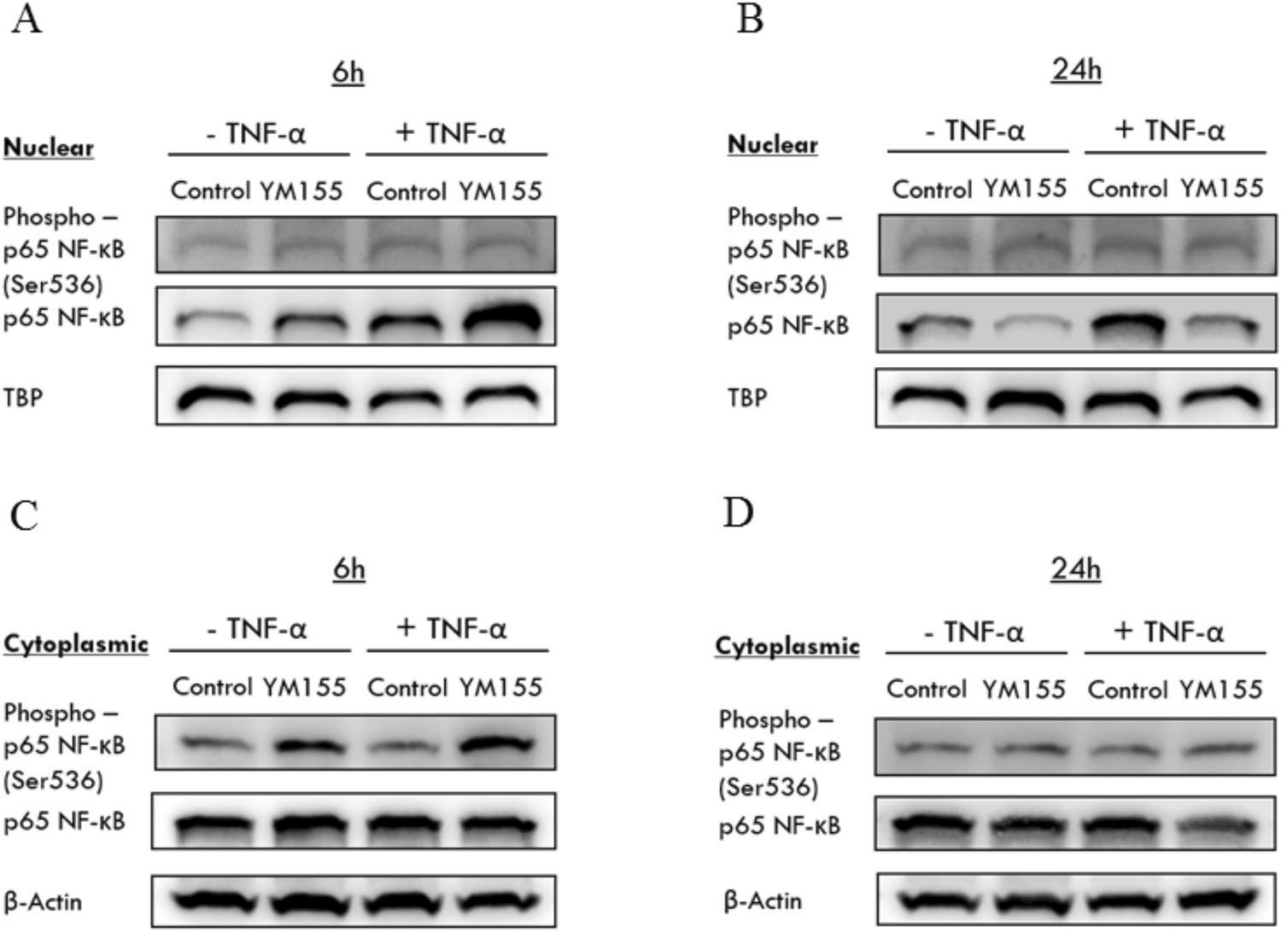
Effects of YM155 on the protein expression of p65 NF-κB. RCC786.0 was treated with 40 nM of YM155 in the absence or presence of TNF-α stimulation. Using western immunoblotting analysis, the expression levels of the phosphorylated (phospho-) and total p65 were probed at 6 h and 24 h. In the nuclear extract of YM155 treated cells, (**A**) there was an increase in p65 NF-κB at 6 h, (**B**) there was a decrease in p65 NF-κB at 24 h. The increased/decreased was greater in cells stimulated with TNF-α. There was no concurrent increase in levels of phosphorylated p65. In the cytoplasmic extract of YM155 treated cells, (**C**) there was an increase in phospho-p65 but total p65 was not affected at 6 h, (**D**) there was no significant change in total p65 at 24 h and phospho- p65 was maintained at basal levels. TATA-box binding protein (TBP) and beta actin (β-actin) were used as loading control for nuclear and cytoplasmic fraction respectively.

Next, we asked if the suppression of NF-κB transcriptional activity in YM155-treated cells could be due to diminished levels of p65/p50 heterodimers, in particular p65 which carries the transactivation domain (TAD) required for transcription. To this end, we monitored the levels of p65 and phosphorylated p65 in the cytosolic and nuclear fractions of treated cells at the 6 h and 24 h time points. These experiments were carried out with or without TNF-α (Fig. 4). Phosphorylation can occur at many serine residues in p65. Here we monitored phosphorylation at Ser536 which is found in TAD. Only phosphorylated p65 will bind to the DNA response elements to initiate transcription.

Figure 4A,C shows the results from YM155-treated cells at the 6 h time point. Levels of cytosolic p65 were unaffected but there were increases in the nuclear levels of p65. This would indicate that YM155 promoted the cytosol-to-nuclear translocation of p65. The increases were more prominent in the presence of TNF-α which might suggest that YM155 potentiated the stimulatory activity of TNF-α. Next we examined the levels of phosphorylated p65 and found elevated levels in the cytosol but not in the nucleus. Similar observations were made in the presence or absence of TNF-α. Thus, we deduced that at the early 6 h time point, YM155 enhanced p65 translocation to the nucleus but did not upregulate p65 transcriptional activity as there was no evidence of increased levels of phosphorylated p65 in the nucleus.

We then examined the effects of YM155 on p65 and phosphorylated p65 levels at the longer 24 h time point. As in the earlier time point, no change was observed in cytosolic p65 but interestingly, there was now a marked decrease in nuclear p65 levels (Fig. 4B,D). Thus, YM155 induced a late suppression of the nuclear translocation of p65. As for phosphorylated p65, these were maintained at basal (low) levels in both cytosolic and nuclear compartments.

Taken together, YM155 has a time dependent effect on the cytosol to nuclear translocation of p65, in which an initial increase in translocation preceded the subsequent decrease. Oddly, the early increase in translocation was not accompanied by enhanced transcriptional activity and this anomaly finds support from the earlier observations on the increased binding of p65 to its consensus sequences but the transcriptional activity remained largely unchanged in luciferase transfected cells (Figs 2 and 3). Possible reasons for the blunted p65 transcriptional response may be that post translational processes (phosphorylation, acetylation) were impeded or that levels of co-activators/co-repressors that are critical for the transcription were lacking. Interestingly, the presence of TNF-α, a strong activator of NF-κB signalling, did not appear to significantly affect the overall process. On the other hand, the late suppression of the cytosol to nuclear translocation of p65 by YM155 did cause the anticipated loss in transcriptional activity, corroborating earlier findings of reduced binding to p65 consensus sequences and a decline in the transcription of affected genes in luciferase transfected cells (but only with TNF-α).

### YM155 activated CYLD activity which led to suppressed IKKβ activity and stabilization of inhibitory IκBα

Thus far, we have shown that YM155 suppressed the nuclear translocation of p65 which led to diminished transcription of NF-κB target genes. This effect was not due to a lack of p65 for translocation as the immunoblots of treated cells revealed levels of cytosolic p65 that were comparable to controls (Fig. 4). mRNA levels of p65 were similarly retained at basal levels upon treatment with YM155 (Supplementary Figure 2). In an earlier investigation, we found that YM155 (40 nM) upregulated cylindromatosis (CYLD) at both the gene and protein level in RCC786.0 cells after 24 h. CYLD is a deubiquitinating enzyme responsible for removing K63-linked polyubiquitin chains from TRAF2. Without its ubiquitin side chain, TRAF2 is unable to activate IKK, which would in turn suppress its ability to phosphorylate IκBα. Consequently, IκBα will retain the NF-κB dimers in their inactive states within the cytosol and prevent their translocation to the nucleus.

**Figure 5:**
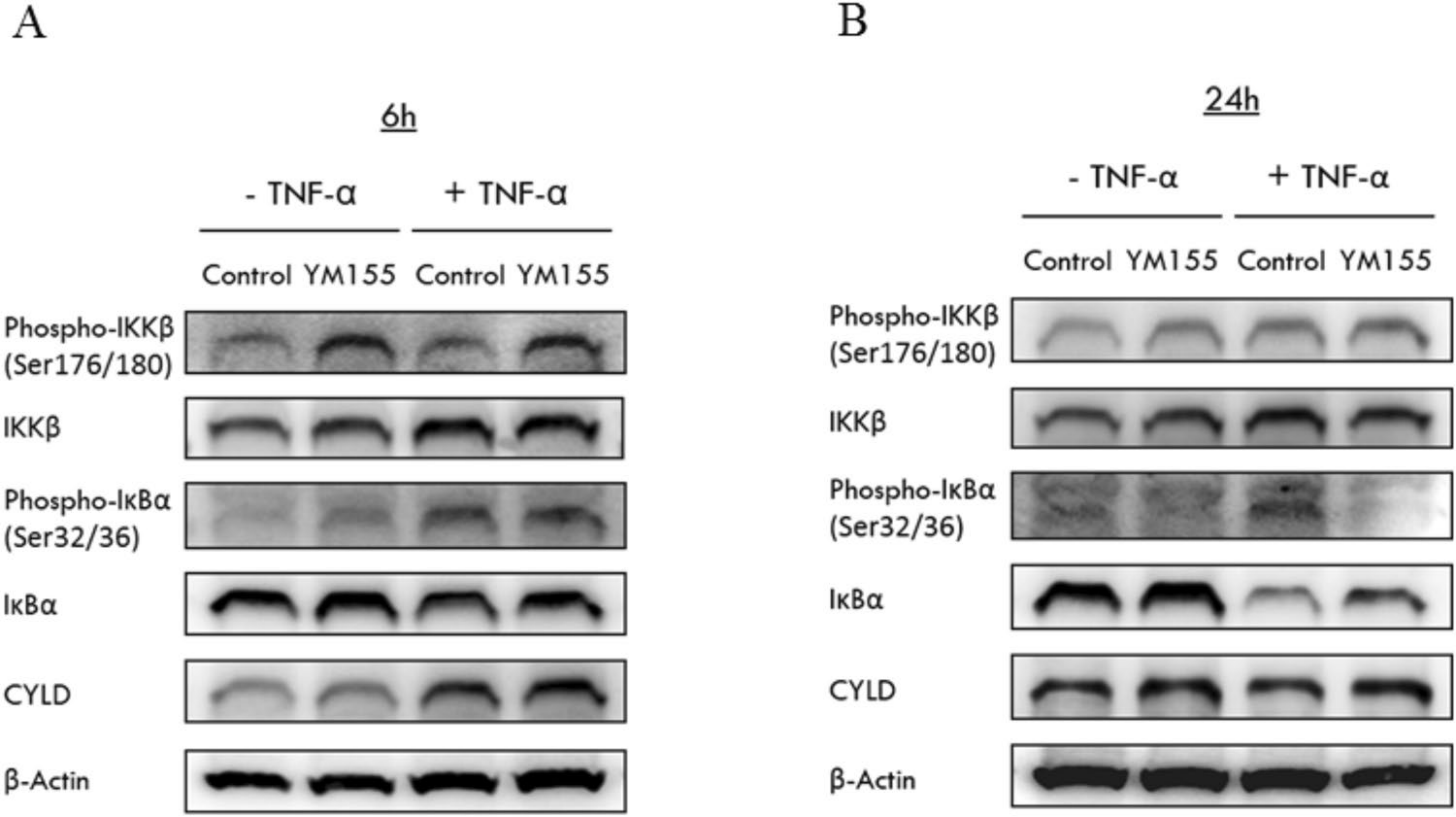
Effects of YM155 on molecules associated with the NF-κB signaling pathway. RCC786.0 was treated with 40 nM of YM155 for 6 h and 24 h in the absence or presence of TNF-α stimulation. Using western immunoblotting analysis, the expression levels of total IKKβ, IκBα and their phosphorylated (phospho-) states and CYLD were probed. (**A**) At 6 h, YM155 increased phospho- IKKβ but other molecules remained largely unchanged compared to control cells. (**B**) At 24 h, YM155 decreased phospho- IκBα, stabilised total IκBα and increased CYLD in the presence of TNF-α stimulation. Beta actin (β-actin) was used as loading control.

To determine if YM155 could have intercepted these upstream events which crucially determine the sequesteration of NF-κB dimers within the cytosol, we examined the levels of IKKβ, phospho-IKKβ, IκBα phospho-IκBα and CYLD in RCC786.0 cells after treatment with YM155 (40 nM) for 24 h. The experiments were repeated in the presence of TNF-α. As shown in Fig. 5, CYLD levels were elevated in the presence of YM155, particularly in cells stimulated with TNF-α (Fig. 5B). IKKβ levels were unchanged but phospho-IKKβ levels were not elevated, which suggests that IKKβ was retained in its inactive state. We found no change or a slight increase in IκBα levels in treated cells and tellingly, negligible phospho-IκBα. Thus, we surmised that inhibitory effects of IκBα on NF-κB would persist. These changes in protein levels support our hypothesis that the stimulatory effects of YM155 on CYLD played a central role in curtailing the nuclear translocation of p65. Augmented CYLD activity suppressed IKKβ which stabilized IκBα and retained NF-κB dimers in their inactive states in the cytosol.

We also probed the levels of CYLD, IKK, phospho-IKK, IκBα and phospho-IkBα at the 6 h time point where there was no suppression of the pathway by YM155. As shown in Fig. 5A, CYLD, IKK and IκBα levels were comparable to those found in control cells. Tellingly, phospho-IKK levels were elevated in the presence of YM155, indicating that IKK was activated and primed to destabilize IκBα. Unfortunately this was not reflected from the protein levels of IκBα and phospho-IκBα which were comparable to controls (Fig. 5A). Nonetheless, the results reiterate the time dependent effect of YM155 on NF-κB signaling, with suppression evident only after a longer 24 h time period.

### YM155 down-regulated survivin transcriptional activity in RCC786.0 cells

**Figure 6:**
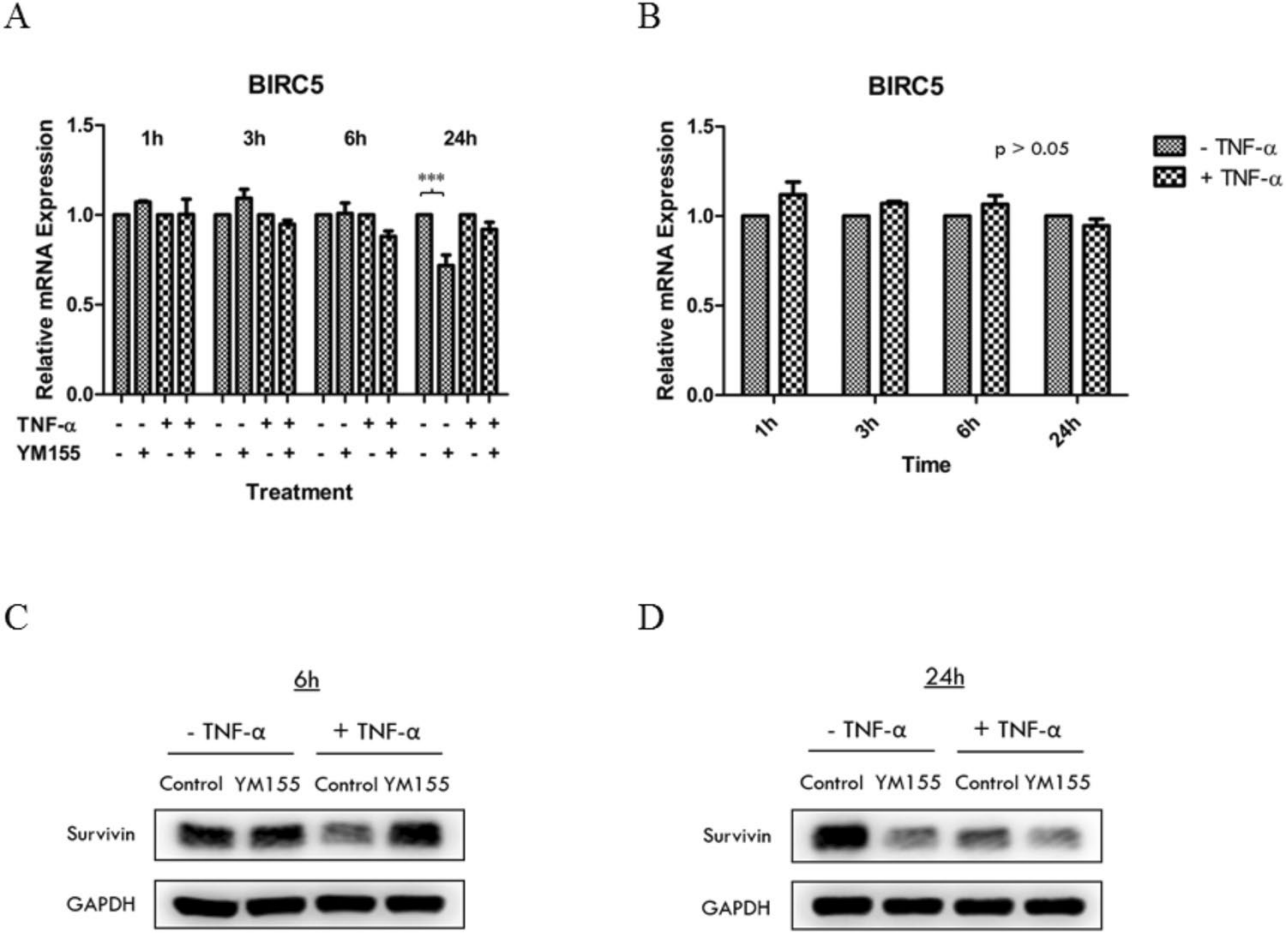
Effects of TNF-α and YM155 on the mRNA and protein expression level of survivin. RCC786.0 was stimulated with 20 ng/ml of TNF-α or/and treated with 40 nM of YM155 for 1 h, 3 h, 6 h and 24 h. The mRNA expression levels of *BIRC5* were determined using qRT-PCR. The signals were normalised to their respective controls (without/with TNF-α and time-points) which is equivalent to 1. (**A**) YM155 induced a significant decrease (~28%) in the mRNA expression of *BIRC5* at 24 h in the absence of TNF-α but not in the presence of TNF-α. (**B**) Stimulation of RCC786.0 with TNF-α did not significantly increase/decrease the mRNA expression of *BIRC5* at all tested time points. ***p < 0.001. The protein expression levels of survivin at (**C**) 6 h and (**D**) 24 h were determined using western immunoblotting analysis. YM155 decreased the protein expression of survivin at 24 h but not at 6 h. Stimulation with TNF-α reduced basal level of survivin protein expression at 6 h and 24 h, making the decrease due to YM155 inhibition less marked.

To further validate our hypothesis that YM155 suppressed NF-κB signaling, we proceeded to demonstrate that suppression extended to genes that are regulated by NF-κB. The gene *BIRC5* which encodes survivin was selected for investigation and the effects of YM155 on the mRNA and protein levels of survivin was examined in Fig. 6. As in the earlier experiments, RCC786.0 cells were treated with 40 nM YM155 and mRNA levels were monitored over time (3 h, 6 h, 24 h) in the presence or absence of TNF-α. We found that YM155 only decreased mRNA levels at the 24 h time point and in the absence of TNF-α. Immunoblotting experiments confirmed the differential loss of survivin at this time point. One difference was that diminished level of survivin was observed with or without TNF-α, unlike mRNA levels where they were apparent only in the absence of TNF-α.

A closer inspection of the survivin immunoblots from control cells exposed to TNF-α showed unusual losses in band intensities at both the 6 h and 24 h time points (Fig. 6C,D). These losses were noticeably absent in control blots derived from cells not treated with TNF-α. While these observations might suggest that TNF-α suppressed survivin expression, it was not supported from the survivin mRNA levels of control cells which were largely the same in the presence or absence of TNF-α. Opposing views have been reported in the literature regarding the effects of TNF-α on survivin expression. Schmidt *et al*. reported upregulated survivin expression in rat dendritic cells stimulated with TNF-α^20^, while others opined that TNF-α had either no effect on survivin mRNA/protein levels or decreased survivin protein levels^21–23^. Notwithstanding these contrarian views, our findings point to the attenuation of survivin expression by YM155 at a late time point, in the absence of TNF-α.

### YM155 reduced the interaction between p65 and its DNA binding sites at the survivin promoter regions

In the preceding sections, we have shown YM155 diminished the binding of p65 to its consensus sequences, attenuated the transcription of NF-κB related genes in treated luciferase transfected RCC786.0 cells, delayed the translocation of p65 to the nucleus and suppressed levels of phosphorylated p65 within that compartment. These events culminated in the YM155-induced attenuation of survivin expression. To provide further collateral evidence to support our hypothesis, we investigated the recruitment of p65 to NF-κB-dependent promoters by the chromatin immunoprecipitation (ChIP) assay. Briefly, this technique is a means of examining the specific association of transcription factors with DNA in the context of living cells. It provides a snapshot of the interaction between the NF-κB heterodimers (p65/p50) and their DNA binding sites on the target gene within the natural chromatin context of the cell. Primers designed to flank the NF-κB DNA binding region were used to amplify the chromatin fragments immunoprecipitated by the p65 antibody. In this manner, the extent to which p65 binds to the *BIRC5* promoter region can be quantified by qRT-PCR.

**Figure 7:**
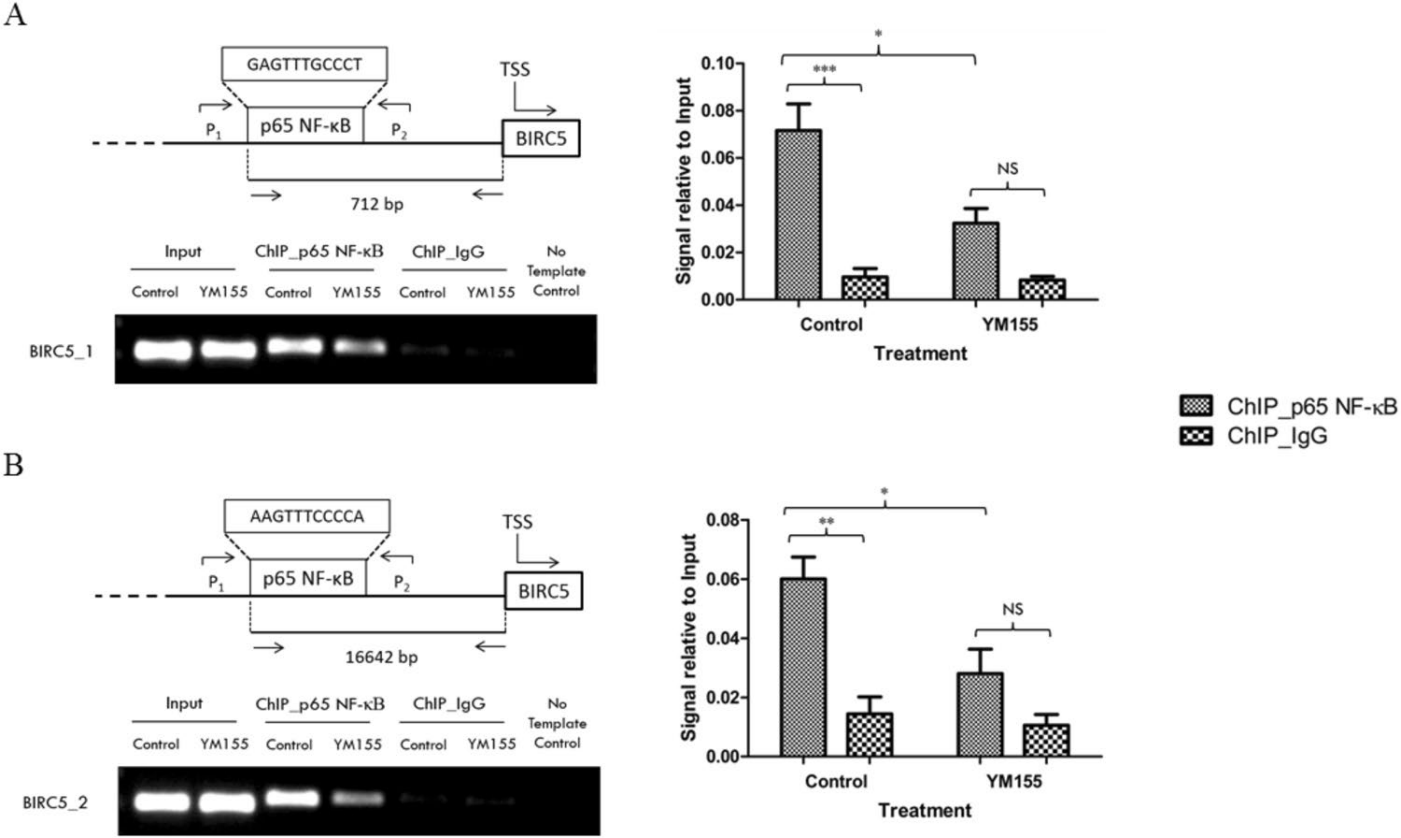
Effects of YM155 on the recruitment of p65 NF-κB to the transcription factor binding sites of the *BIRC5* promoter regions. RCC786.0 was treated with 40 nM of YM155 for 24 h. Chromatin fragments were immunoprecipitated using the p65 NF-κB antibody and its corresponding IgG was used as a control. Input consists of chromatin fragments that were not subjected to immunoprecipitation. Purified chromatin was amplified using conventional PCR and gel electrophoresis of the amplified fragments was performed. The degree of interaction between p65 NF-κB and its binding sites of the respective genes was quantitated using qRT-PCR and the signals were normalised to their respective inputs. A decrease in *BIRC5* band intensity was observed in YM155 treated cells and qRT-PCR analysis indicated a 2 fold reduction of p65 NF-κB on **(A)** binding site 1 (BIRC5_1) and **(B)** binding site 2 (BIRC5_2) of the *BIRC5* promoter region. *p < 0.05 from its untreated control and **p < 0.01 and ***p < 0.001 from its corresponding IgG. NS, Not significant; P1, forward primer; P2, reverse primer; TSS, transcription start site.

RCC786.0 cells were first treated with 40 nM of YM155 for 24 h and fixed with formaldehyde for analysis by ChIP. We found that treatment with YM155 led to a reduction (approximately 2-fold) in the binding of p65 to two separate sites of the *BIRC5* promoter region (Fig. 7A,B). These observations recapitulate the gene expression results described in the preceding section where YM155 (40 nM, 24 h) decreased mRNA and protein levels of survivin.

## Discussion

YM155 has been a highly controversial anticancer agent since it was reported nearly a decade ago. Proclaimed as the first small molecule to suppress survivin, it soon became apparent that YM155 had pleiotropic effects, affecting diverse targets such as DNA^24^, and mitochondria as well as processes of which autophagy and the ER stress response have been implicated^11^. We have shown here that YM155 suppressed the NF-κB pathway through a sequence of events that culminated in the interception of the nuclear translocation of p65/p50. We proposed that the activation of CYLD by YM155 provided the initial trigger which led to the inhibition of IKKβ, thereby stabilizing IκBα in its inhibitory state and the retention of NF-κB dimers in the cytosol. Consequently, the transcriptional competency of these heterodimers was suppressed and we have assembled a body of collateral evidence to support this claim. Accordingly, we demonstrated that YM155 diminished the binding of p65 to DNA consensus sequences, decreased NF-κB transcriptional activity of luciferase transfected cells and suppressed levels of p65 in the nuclear compartment, and through the ChIP assays, provided evidence of diminished binding between p65 and the promoter regions of survivin in YM155 treated cells.

It should be highlighted that the suppressive effects of YM155 on the NF-κB pathway was only evident at 24 h and not at the earlier 6 h time point, where YM155 had an anomalous stimulatory effect on the pathway. Tellingly, we have earlier shown that in YM155 treated RCC786.0 cells, CYLD mRNA levels were upregulated at the 24 h time point but returned to basal levels after 48 h^13^. It is conceivable that had we monitored the effects of YM155 for a longer time period (>24 h), we would have obtained different results. In fact, we speculate that time dependency in the effects of YM155 may explain the contrarian results of a related study on the interaction of YM155 with the NF-κB pathway. In that report, results were gathered at the 48 h time point from YM155-treated non-small cell lung cancer H1299 cells^14^. Although the authors concluded that YM155 also suppressed the NF-κB pathway, its effects were directed against the phosphorylation of p50 rather than the nuclear translocation of NF-κB dimers. That the H1299 cells possess mutated p53, unlike RCC786.0 cells which carry wild type p53, may yet be another confounding factor.

Unlike the suppressive effects of YM155, evidence to support its early stimulatory activity on the NF-κB pathway was not compelling. Notably, increases in the binding affinity of p65 to its DNA consensus sequences and elevated levels of p65 in the nucleus did not translate to greater transcriptional activity. We speculate that YM155 may have impeded post translational processes or adversely affected the levels of co-activators/ co-repressors that are required for the transcription process.

To further validate our hypothesis that YM155 suppressed NF-κB signaling, we proceeded to demonstrate that suppression extended to genes that are regulated by NF-κB. The gene *BIRC5* which encodes survivin was selected for investigation and we showed that YM155 downregulated *BIRC5* expression, reduced survivin levels and intercepted the interaction between p65 and the DNA binding sites on the promoter regions of survivin. Tellingly, the reductions in the *BIRC5* mRNA and protein levels of survivin in YM155 treated cells were only evident at 24 h, at which point, the suppressive effects of YM155 on nuclear p65 were also observed. If the NF-κB modulatory effects of YM155 were related to indirect inhibition of survivin, changes in survivin levels would precede those involved in the NF-κB pathway, which were not observed here. These misaligned time-related trends, together with the stimulatory effects of YM155 on CYLD, led us to propose NF-κB involvement in YM155-induced suppression of survivin, although we cannot completely discount the contributions of other signaling pathways like p53 at this stage of our investigations.

Another point that warrants mention is the influence of TNF-α on the effects of YM155. Broadly similar results were obtained regardless of whether cells were treated with TNF-α or not, although some effects were more marked in the presence of TNF-α. Taken together, we propose that the stimulatory effects of TNF-α on the NF-κB pathway were not affected by YM155.

In conclusion, we have shown the time dependent (24 h) suppression of NF-κB signaling by YM155 in RCC786.0 cells which occurred independently of TNF-α stimulation. The suppression may be due to diminished ubiquitination induced by greater CYLD activity. Given that NF-κB dysfunction is central to oncogenic processes; it has become a target of interest for anti-cancer treatment^25^ with reported promising therapeutic effects^26,27^. The suppressive effects of YM155 on NF-κB signaling provide a wider perspective on the pharmacodynamic effects elicited by this unusual molecule and potentially, its use in therapeutic combinations to enhance the clinical efficacy in the treatment of RCC and other cancers.

## Materials and Methods

### Cell culture

RCC786.0 was obtained from American Type Culture Collection (ATCC, Manassas, VA, USA). All cells were maintained in high glucose Dulbecco’s Modified Eagle Medium (DMEM) containing L-glutamine, sodium pyruvate, 10% FBS and 100U Penicillin/Streptomycin (Gibco, Waltham, MA, USA). All cells were grown in a humidified atmosphere of 5% CO_2_ and 95% filtered atmospheric air and maintained at a temperature of 37 ^o^C.

### NF-κB binding activity

RCC786.0 cells were treated with increasing concentrations of YM155 (20–160 nM) or 0.1% DMSO in the presence or absence of TNF-α (20 ng/ml) for 6 h or 24 h. NE-PER Nuclear & Cytoplasmic Extraction Reagents (Thermo Fisher Scientific, Waltham, MA, USA) was used to lyse and extract the nuclear and cytoplasmic protein fraction of cells. The nuclear fractions of the cells were added to a streptavidin-coated 96-well plate with bound NF-κB biotinylated-consensus sequence provided in the NF-κB p65 Transcription Factor Kit (Thermo Fisher Scientific, Waltham, MA, USA) and subsequently processed following manufacturer instructions. The relative NF-κB DNA binding activity of treated cells was determined by comparing with the chemiluminescent signal obtained from control cells.

### NF-κB luciferase reporter assay

A firefly luciferase reporter vector which contains five copies of NF-κB response elements, pGL4.32[luc2P/NF-κB-RE/Hydro] and a renilla luciferase vector which is a control reporter, pGL4.74[hRluc/TK] were used (Promega Corporation, Fitchburg, WI, USA). RCC786.0 cells were transiently co-transfected with both vectors using Lipofectamine® Transfection Reagent (Invitrogen, Waltham, MA, USA). Twenty-four hours post-transfection, the cells were treated with 40 nM of YM155 or 0.1% DMSO in the absence or presence of TNF-α (20 ng/ml) for 6 h or 24 h. Dual-Glo® Luciferase Assay System (Promega, Corporation, Fitchburg, WI, USA) was used to measure the luciferase activities. Transcription activity of NF-κB was calculated as the relative firefly luciferase activity (experimental reporter) normalized to the renilla luciferase activity (control reporter). Changes in transcription activity of NF-κB of treated samples were expressed as fold change with respect to the corresponding control samples.

### Protein analysis using western immunoblotting

To ensure preservation of protein phosphorylation and high total protein yields, cells were lysed directly in M-PER Mammalian Protein Extraction Reagent (Pierce, Waltham, MA, USA) supplemented with Halt Protease and Phosphatase Inhibitors (Pierce, Waltham, MA, USA). When nuclear and cytoplasmic protein fractions of cells were required, NE-PER Nuclear & Cytoplasmic Extraction Reagents (Thermo Fisher Scientific, Waltham, MA, USA) was used. Quantitation of proteins was made using the bicinchoninic acid (BCA) method (Pierce, Waltham, MA, USA). Proteins were electrophoresed by SDS-PAGE and transferred onto a nitrocellulose membrane. Blots were incubated with the indicated primary antibodies (Cell Signaling Technology Inc, Danvers, MA, USA) and horse-radish peroxidase conjugated secondary antibodies. All proteins were visualised with Immobilon chemiluminescent detection reagent (Millipore, Billerica, MA, USA).

### Polymerase chain reaction and gel electrophoresis

Nested PCR was performed using two sets of primers (Supplementary Table 1). Conditions for the PCR reaction included an initial incubation step of 15 minute at 95 °C and 30 cycles (1^st^ PCR) or 15 cycles (2^nd^ PCR) of 30 seconds at 94 °C, 45 seconds at 55 °C and 60 seconds at 72 °C. A final extension step of 10 minutes was performed at 72 °C. Amplified DNA fragments were resolved by gel electrophoresis, stained with gel red and visualised under UV light.

### Quantitative real time polymerase chain reaction

Express SYBR® GreenER™ qPCR SuperMix Universal (Invitrogen, Waltham, MA, USA) and TaqMan® Fast Advanced Master Mix (Applied Biosystems, Waltham, MA, USA) were used in the detection of PCR products in real time. qRT-PCR was performed using specific primers and DNA probes (Supplementary Tables 1 and 2). Conditions for the SYBR green method were 50 °C for 2 minutes, 95 °C for 2 minutes and then 40 cycles, each consisting of 30 seconds at 94 °C, 45 seconds at 55 °C and 45 seconds at 72^o^C. The amount of DNA in each unknown sample was extrapolated from standard curves for each PCR reaction, by making 10-fold serial dilutions covering the concentration range equivalent to 10 to 0.001 ng DNA. Conditions for the Taqman method were 2 minutes at 50 °C, 20 seconds at 95 °C and then 40 cycles, each consisting of 3 seconds at 95 °C and 30 seconds at 60 °C. The housekeeping gene used was glyceraldehyde-3-phosphate dehydrogenase (GAPDH). Comparative Ct method was used to quantify the expression of the gene of interest.

### Chromatin Immunoprecipitation (ChIP)

ChIP-IT High Sensitivity kit (Active Motif, Carlsbad, CA, USA) was used to determine the association of transcription factor p65 NF-κB with its specific genomic regions on the survivin gene *BIRC5*. RCC786.0 cells were treated with 40 nM of YM155 or 0.1% DMSO (control) for 24 h and subjected to cell fixation, chromatin sonication, immunoprecipitation and DNA purification. Rabbit IgG was used as a negative control in the immunoprecipitation experiments. The immunoprecipitated fraction was analysed by PCR and qRT-PCR to determine the abundance of the target DNA sequence(s) relative to the input chromatin.

### Statistical analysis

The graphing and statistical analysis software GraphPad Prism version 5.0 (GraphPad Software Inc., San Diego, CA, USA) and Excel (Microsoft Corporation, Redmond, WA, USA) were used to plot and analyse data. Graphs were plotted to show mean values and error bars which depicted standard error of the mean (SEM). The student’s t-test and analysis of variance (ANOVA) with Bonferroni post–hoc test were used for the comparison of mean values between two and multiple (> 2) groups, respectively. A minimum of 95% level of significance (p < 0.05) was used to define statistical significance. All experiments were repeated at least twice.

## Electronic supplementary material

Supplementary Information 

## References

[1] Griffin, N., Gore, M. E. & Sohaib, S. A. Imaging in metastatic renal cell carcinoma. *AJR Am J Roentgenol* **189**, 360–370, 189/2/360 [pii]10.2214/AJR.07.2077 (2007). 10.2214/AJR.07.2077 17646462

[2] Motzer RJ (2003). Prognostic factors and clinical trials of new agents in patients with metastatic renal cell carcinoma. Crit Rev Oncol Hematol.

[3] Nakahara T (2011). Broad spectrum and potent antitumor activities of YM155, a novel small-molecule survivin suppressant, in a wide variety of human cancer cell lines and xenograft models. Cancer Sci.

[4] Tolcher AW (2008). Phase I and pharmacokinetic study of YM155, a small-molecule inhibitor of survivin. J Clin Oncol.

[5] Clemens MR (2015). Phase II, multicenter, open-label, randomized study of YM155 plus docetaxel as first-line treatment in patients with HER2-negative metastatic breast cancer. Breast Cancer Res Treat.

[6] Sasaki R, Ito S, Asahi M, Ishida Y (2015). YM155 suppresses cell proliferation and induces cell death in human adult T-cell leukemia/lymphoma cells. Leuk Res.

[7] Nakahara T (2007). YM155, a novel small-molecule survivin suppressant, induces regression of established human hormone-refractory prostate tumor xenografts. Cancer Res.

[8] Iwasa T (2008). Radiosensitizing effect of YM155, a novel small-molecule survivin suppressant, in non-small cell lung cancer cell lines. Clin Cancer Res.

[9] Zhang W (2016). Targeting of Survivin Pathways by YM155 Inhibits Cell Death and Invasion in Oral Squamous Cell Carcinoma Cells. Cell Physiol Biochem.

[10] Carew JS (2015). Targeting Survivin Inhibits Renal Cell Carcinoma Progression and Enhances the Activity of Temsirolimus. Mol Cancer Ther.

[11] Jane EP, Premkumar DR, Sutera PA, Cavaleri JM, Pollack IF (2017). Survivin inhibitor YM155 induces mitochondrial dysfunction, autophagy, DNA damage and apoptosis in Bcl-xL silenced glioma cell lines. Molecular carcinogenesis.

[12] Rauch A (2014). Survivin and YM155: how faithful is the liaison?. Biochim Biophys Acta.

[13] Sim MY, Huynh H, Go ML, Yuen JSP (2017). Action of YM155 on clear cell renal cell carcinoma does not depend on survivin expression levels. PLoS One.

[14] Ho SH, Ali A, Chin TM, Go ML (2016). Dioxonaphthoimidazoliums AB1 and YM155 disrupt phosphorylation of p50 in the NF-kappaB pathway. Oncotarget.

[15] Dey A, Tergaonkar V, Lane DP (2008). Double-edged swords as cancer therapeutics: simultaneously targeting p53 and NF-kappaB pathways. Nat Rev Drug Discov.

[16] Schneider G (2010). Cross talk between stimulated NF-kappaB and the tumor suppressorp53. Oncogene.

[17] Schneider G, Kramer OH (2011). NFkappaB/p53 crosstalk-a promising new therapeutic target. Biochim Biophys Acta.

[18] Guo C (2009). 9-Aminoacridine-based anticancer drugs target the PI3K/AKT/mTOR, NF-kappaB and p53 pathways. Oncogene.

[19] Gupta SC, Sundaram C, Reuter S, Aggarwal BB (2010). Inhibiting NF-kappaB activation by small molecules as a therapeutic strategy. Biochim Biophys Acta.

[20] Schmidt SM (2003). Survivin is a shared tumor-associated antigen expressed in a broad variety of malignancies and recognized by specific cytotoxic T cells. Blood.

[21] O’Connor DS (2000). Control of apoptosis during angiogenesis by survivin expression in endothelial cells. Am J Pathol.

[22] Liu F (2011). TNFalpha cooperates with IFN-gamma to repress Bcl-xL expression to sensitize metastatic colon carcinoma cells to TRAIL-mediated apoptosis. PLoS One.

[23] Gordon GJ (2007). Inhibitor of apoptosis proteins are regulated by tumour necrosis factor-alpha in malignant pleural mesothelioma. J Pathol.

[24] Glaros TG (2012). The “survivin suppressants” NSC 80467 and YM155 induce a DNA damage response. Cancer Chemother Pharmacol.

[25] Baud V, Karin M (2009). Is NF-kappaB a good target for cancer therapy? Hopes and pitfalls. Nat Rev Drug Discov.

[26] Nunes, J. J., Pandey, S. K., Yadav, A., Goel, S. & Ateeq, B. Targeting NF-kappa B Signaling by Artesunate Restores Sensitivity of Castrate-Resistant Prostate Cancer Cells to Antiandrogens. *Neoplasi*a **19**, 333–345, S1476-5586(16)30358-X [pii] 10.1016/j.neo.2017.02.002 (2017). 10.1016/j.neo.2017.02.002 PMC5358938 28319807

[27] Tornatore L (2014). Cancer-selective targeting of the NF-kappaB survival pathway with GADD45beta/MKK7 inhibitors. Cancer Cell.

