# Supplementary material for: Anti-survivin effect of the small molecule inhibitor YM155 in RCC cells is mediated by time-dependent inhibition of the NF-κB pathway
Source: Sci Rep. 2018 Jul 6;8:10289. doi: 10.1038/s41598-018-28213-3 (PMC6035265; doi:10.1038/s41598-018-28213-3)

Anti-survivin effect of the small molecule inhibitor YM155 in RCC cells is mediated by time-dependent inhibition of the NF-κB pathway

Mei Yi Sim*[1,2], John Shyi Peng Yuen[2] and Mei Lin Go[1]

[1] Department of Pharmacy, National University of Singapore, 18 Science Drive 4, 117543, Republic of Singapore

[2] Department of Urology, Singapore General Hospital, 20 College Road, 169856, Republic of Singapore

Supplementary Figure 1

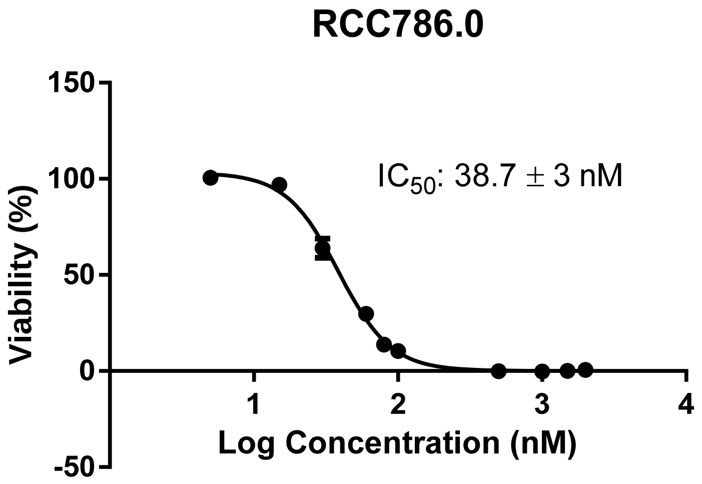

Supplementary Table 1

Name	Forward Primer	Reverse Primer
BIRC5_1_External	CCCAGCTCCAGAAGTGACTC	ACTGGGCCACTACCGTGATA
BIRC5_1_Internal	AACACCCTGTTCCAAAGCAG	GGGCCACTACCGTGATAAGA
BIRC5_2_External	AGATGGCTTTCAGCAAAGGA	GACTCCCTACAGCCACAAGC
BIRC5_2_Internal	GGGCTTATCAGACACCAACC	TAACACGCTCACATCCATCC

Supplementary Table 2

Gene Symbol	Assay ID	Context Sequence
BIRC5	Hs00153353_m1	CCAAGAACAAAATTGCAAAGGAAAC
CYLD	Hs00211000_m1	CGGGATGGTGGTCAGAATGGCTTCA
RelA	Hs00153294_m1	AGTACCTGCCAGATACAGACGATCG
GAPDH	Hs02758991_g1	GACTCATGACCACAGTCCATGCCAT

Supplementary Figure 2

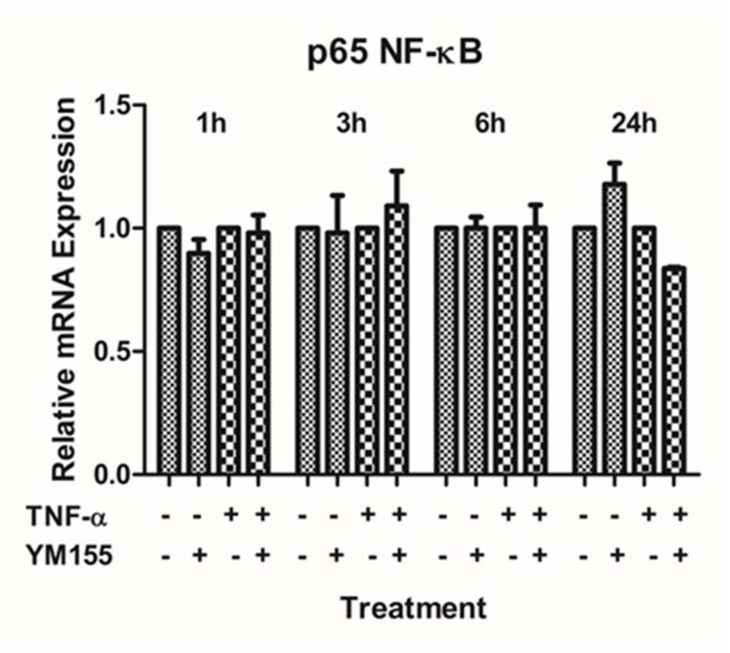

Supplement: Supplementary file 1 — Supplementary Information [file 41598_2018_28213_MOESM1_ESM.pdf]
